# Biomechanical Comparison of Fixation Methods for Posterior Wall Fractures of the Acetabulum: Conventional Reconstruction Plate vs. Spring Plate vs. Variable Angle Locking Compression Plate

**DOI:** 10.3390/medicina60060882

**Published:** 2024-05-28

**Authors:** HoeJeong Chung, Hoon-Sang Sohn, Jong-Keon Oh, Sangho Lee, DooSup Kim

**Affiliations:** 1 Department of Orthopedic Surgery, Wonju College of Medicine, Yonsei University, Wonju Severance Christian Hospital, 20, Ilsan-Ro, Wonju 26426, Republic of Korea; hjchung29@yonsei.ac.kr (H.C.); traumasohn@gmail.com (H.-S.S.); sangholee9@naver.com (S.L.); 2 Department of Orthopaedic Surgery, Korea University Guro Hospital, Korea University Medicine, 148, Gurodong-Ro, Guro-Gu, Seoul 08308, Republic of Korea; jkoh@korea.ac.kr

**Keywords:** biomechanics, acetabulum, posterior wall fracture, variable angle plate

## Abstract

*Background and Objectives*: Acetabular fractures, though infrequent, present considerable challenges in treatment due to their association with high-energy trauma and poor prognoses. Posterior wall fractures, the most common type among them, typically have a more favorable prognosis compared to other types. Anatomical reduction and stable fixation of the posterior wall are crucial for optimal treatment outcomes. This study aimed to biomechanically compare three commonly used fixation methods for posterior wall fractures of the acetabulum—a conventional reconstruction plate, a spring plate, and a 2.7 mm variable angle locking compression plate (VA-LCP). *Materials and Methods*: The study utilized 6 fresh-frozen cadavers, yielding 12 hemipelvises free from prior trauma or surgery. Three fixation methods were compared using a simple acetabulum posterior wall fracture model. Fixation was performed by an orthopedic specialist, with prebending of plates to minimize errors. Hemipelvises were subjected to quasi-static and cyclic loading tests, measuring fracture gap, stiffness, and displacement under load. *Results*: It showed no significant differences in fracture gap among the three fixation methods under cyclic loading conditions simulating walking. However, the conventional reconstruction plate exhibited a greater stiffness compared to the spring and variable angle plates. Fatigue analysis revealed no significant differences among the plates, indicating a similar stability throughout cyclic loading. Despite differences in stiffness, all three fixation methods demonstrated adequate stability under loading conditions. *Conclusions*: While the conventional reconstruction plate demonstrated a superior stiffness, all three fixation methods provided sufficient stability under cyclic loading conditions similar to walking. This suggests that postoperative limitations are unlikely with any of the three methods, provided excessive activities are avoided. Furthermore, the variable angle plate—like the spring plate—offers an appropriate stability for fragment-specific fixation, supporting its use in surgical applications. These findings contribute to understanding the biomechanical performance of different fixation methods for acetabular fractures, facilitating improved surgical outcomes in challenging cases.

## 1. Introduction

Acetabular fractures are relatively uncommon but present significant treatment challenges, primarily due to their association with high-energy trauma, often resulting in poor prognoses [1,2]. Among the various classifications, posterior wall fractures are the most prevalent and are generally associated with a relatively simpler treatment approach and better outcomes [3,4]. These fractures can be managed either non-surgically or surgically, depending on the stability and congruence of the fracture. Non-surgical treatment is considered for stable, congruent posterior wall fractures [5], whereas surgical intervention is recommended when fractures lead to hip joint instability or incongruity [6]. According to Moed et al., posterior wall fragments that comprise more than 50% of the hip joint surface on a CT scan are deemed unstable [7,8]. For borderline cases, examination under anesthesia (EUA) is utilized to assess the stability and to determine the necessity for surgery [9]. The standard surgical approach typically involves open reduction and internal fixation (ORIF), with total hip arthroplasty being an option for severely comminuted fractures in elderly patients [2,10]. Achieving an anatomical reduction and stable fixation of the posterior wall is crucial for successful outcomes [10,11,12].

Various techniques for fixing posterior wall fractures have been developed, including the use of plates, lag screws, and spring plates [13,14,15,16,17]. Locking compression plates (LCPs) are gaining favor in orthopedic trauma treatments due to their enhanced stability and the presumed benefits in osteopenic bone. These plates reduce the need for lag screws, thus mitigating the risk of intra-articular penetration [18,19,20,21,22]. However, they may pose challenges in managing small peripheral or comminuted fracture fragments due to potential joint penetration post-fixation [23].

Spring plates have proven effective in managing marginal fractures of the posterior wall, as they provide adequate stability without necessitating extensive dissection like larger reconstruction plates [24]. Although spring plates alone do not increase the stiffness of the fixation, they do improve the ultimate yield strength, making them a viable option for marginal and/or comminuted fragments that are unsuitable for lag screw fixation [25].

For superior dome or comminuted posterior wall fractures, the fragment-specific fixation technique using 2.7 mm VA LCP plates represents a promising alternative. This method provides the stable fixation of small fracture fragments, eliminating the need for an overlapping reconstruction plate. Yet, it carries a potential risk of screw joint penetration in peripheral fractures; further biomechanical evaluation of this technique is needed [26].

Recent clinical reports have underscored the efficacy of the 2.7 mm VA LCP for fragment-specific fixation. Research by Cho et al. highlights the advantages of the variation angle LCP plate in multifragmentary fractures, such as improved positioning of each fragment, reduced soft tissue damage, and enhanced fixation of challenging areas, including the superior dome [26]. Nonetheless, more biomechanical studies are required to fully understand the mechanics of fracture fixation. Clinical trials are crucial, but laboratory tests, including comparative studies using saw bone models and cadaver studies for biocompatibility, also play an essential role in this domain [27,28,29,30].

This study aims to perform a mechanical analysis of different fixation plates using a posterior wall fracture model from a cadaveric hemi-pelvis, with a particular focus on evaluating the stability of the 2.7 mm VA-LCP plate, which has been the subject of less clinical research compared to other plates.

## 2. Materials and Methods

The study utilized 6 fresh frozen cadavers, yielding 12 hemipelvises, free from trauma, surgery, or metabolic bone diseases. The cadavers, averaging 62.5 years of age (ranging from 53 to 78), consisted of 5 males and 1 female. Stored at −20 °C, the cadavers were thawed at room temperature 24 hours prior to the experiment. After removing surrounding muscles and soft tissues from the pelvis, the bones were cleaned using acetone. The cadavers used were ethically sourced, each donated with informed consent from tertiary care hospitals, in strict accordance with national legal and ethical standards.

**Figure 1:**
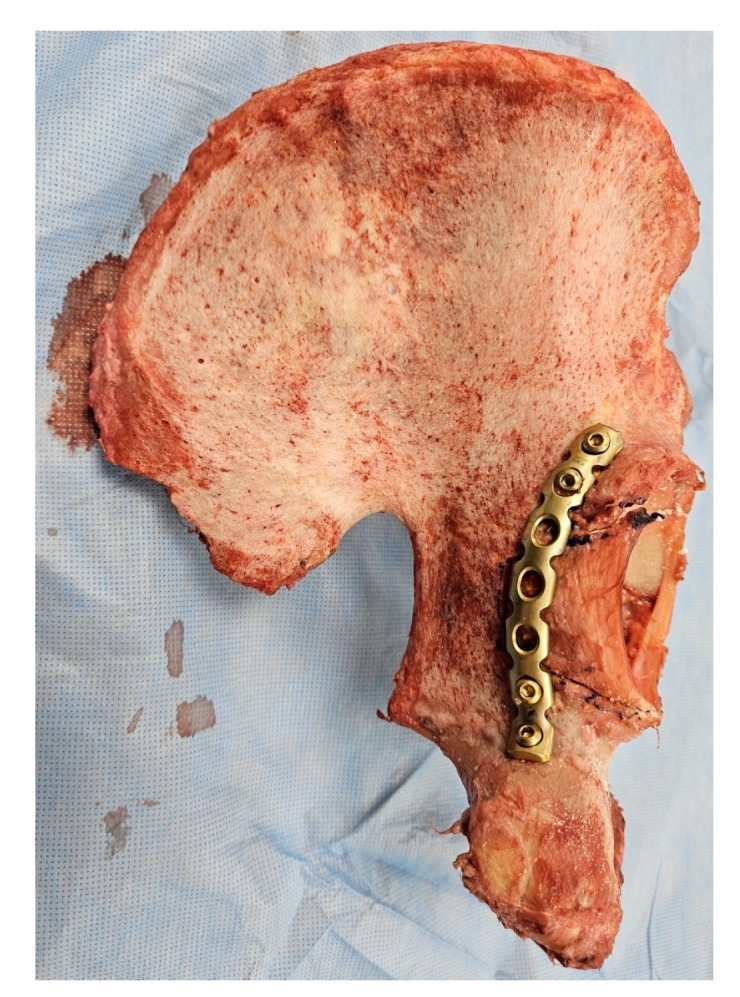
Experimental gross photo of cadaveric posterior fracture wall model fixed with reconstruction plate.

**Figure 2:**
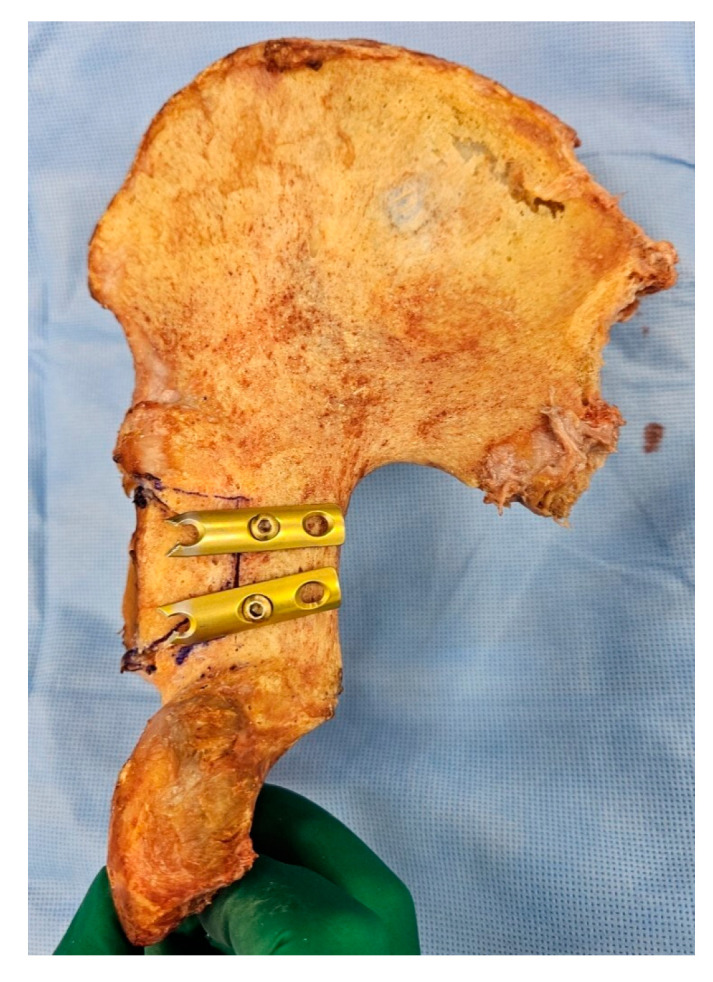
Experimental gross photo of cadaveric posterior fracture wall model fixed with two spring plates.

**Figure 3:**
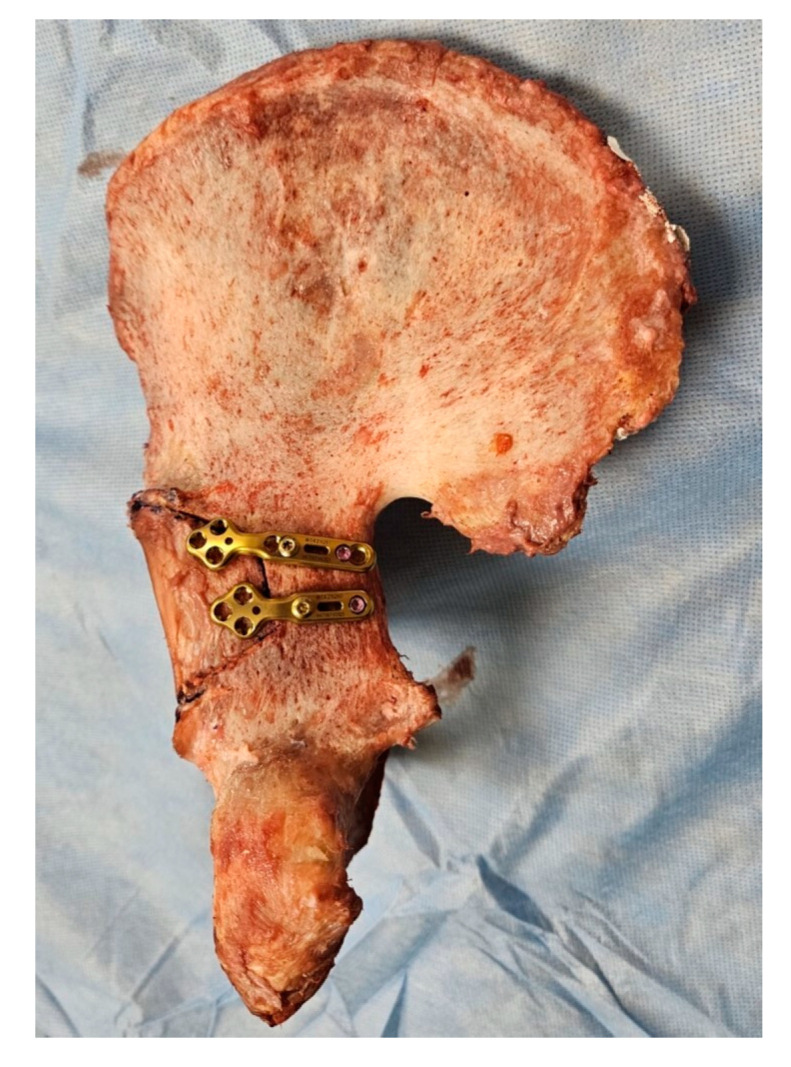
Experimental gross photo of cadaveric posterior fracture wall model fixed with two variable angle plates.

For the posterior fracture wall model, three fixation methods were compared. The first method involves fixation using a reconstruction plate with 3.5 mm cortical screws [22]. The second method uses two spring plates. We cut 1/3 of a semitubular plate and took a four-hole one-third tubular plate. First, we cut off the tip through a hole and bent the newly created prongs downwards to create small hooks, fixing it with 3.5 mm cortical screws [24,31]. The third method employs fixation using two 2.7 mm VA LCPs and 2.7 mm locking screws (Figure 1, Figure 2 and Figure 3) [26]. The plates used are as follows: titanium 3.5 mm LCP Reconstruction Plate, DePuy Synthes, Basel, Switzerland; titanium 3.5 mm 1/3 Tubular Plate, DePuy Synthes, Basel, Switzerland; and Titanium 2.7 mm VA-LCP Cloverleaf fusion plate, DePuy Synthes, Basel, Switzerland. Four hemipelves were used for each type of plate.

**Figure 4:**
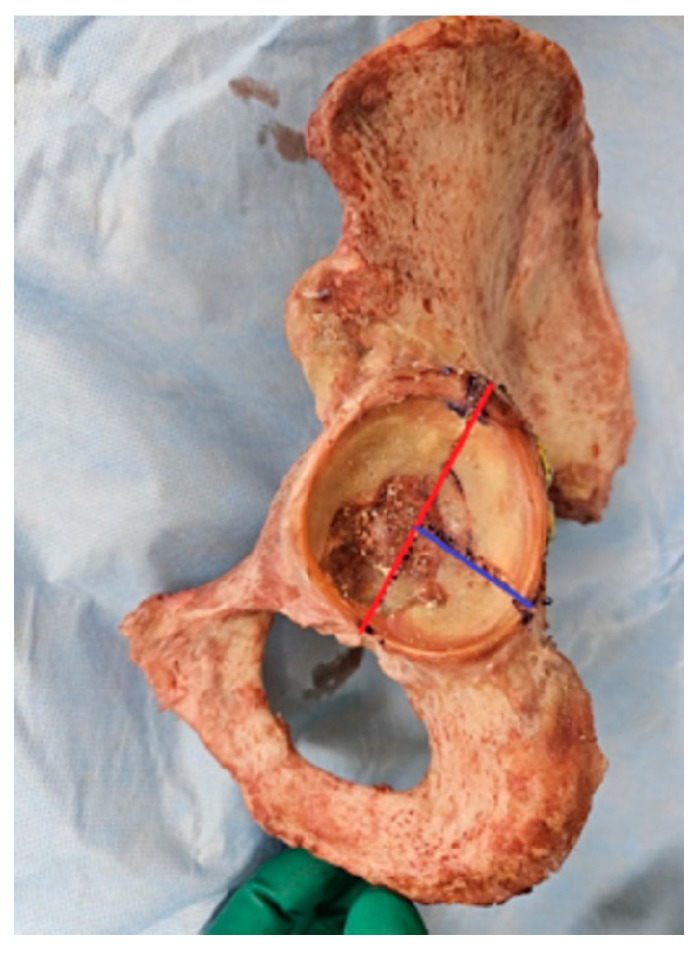
Posterior wall fracture model of study. We set up the transverse ligament as 0 degrees (for use as a reference point (red line)) and then considered the acetabular posterior area at a positive angle. We made a fracture model using a linear saw by setting the point corresponding to 90 degrees as the most common area for posterior wall fractures (blue line).

The fracture model employed was a simple acetabulum posterior wall fracture, not considering marginal impaction (AO/OTA classification 62-A1.1) [32]. According to Cho et al. (who investigated the mapping of acetabular posterior wall fractures using three-dimensional virtual reconstruction software: Mimics Medical 21.0 version software, Materialise, Leuven, Belgium and 3-matic Med ical 13.0 version software, Materialise, Leuven, Belgium), when viewing the acetabular intraarticular portion as a two-dimensional circle, setting the transverse ligament at 0 degrees, and then considering the acetabular posterior area at a positive angle, the fracture angle 90 degrees included angles from 6.2 to 96.3 degrees. The ratio of the fracture angle 90 degrees and the fragment including the acetabulum rim to the longest part of the acetabulum outer cortex, termed as the ‘fracture span’, is described. (Figure 4) The shape with a fracture span of 0.65 is reported to be the most common type of posterior wall fracture [33]. Based on this study, we created our posterior wall fracture model. We marked the fracture line on the acetabulum with a pen and created the most common type of posterior wall fracture model using a linear saw.

**Figure 5:**
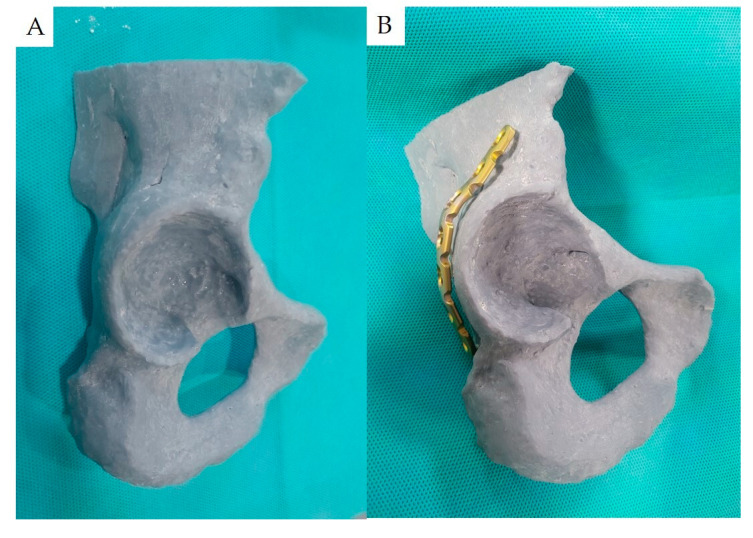
(**A**) Three-dimensional printed anatomical models for planning and surgery simulation. (**B**) Prebending of plate for each hemipelvis was performed to minimize errors related to the inherent fixation and to provide stability.

Fixation was performed using reduction forceps and pointed ball tip pushers to maintain reduction, keeping the fracture gap within 2 mm. All fixation methods were conducted by an orthopedic specialist. Three-dimensional-printed anatomical models for planning and surgery simulation, patient-specific instruments (PSI), generation of prostheses with 3D-additive manufacturing, and custom 3D-printed prostheses were used. Pre-bending for each hemipelvis was performed using 3D modeling to minimize errors related to the inherent fixation strength of the plate. Also, proper bending is important to provide sufficient stability [34,35]. The pre-bending of the reconstruction plate and LCP VA plate, respectively, produced a 3D-printed model and was also performed by the same specialist to reduce bias. (Figure 5) We used a 3D printer (Sindoh A1 SD, Sindoh Co., Ltd, Seoul, South korea, and S-Plastic Model 2.0, Graphy, Seoul, South Korea) as materials.For representing 3D bone models, Mimics Medical 21.0 version software, Materialise, Leuven, Belgium and 3-matic Medical 13.0 version software, Materialise, Leuven, Belgium were used.

The posterior wall model was attached to a specially made jig, in which the hemipelvis fit. The jig was designed to apply loading in a direction perpendicular to the plane connecting the iliac spine and the symphysis pubis, reflecting the direction of maximum loading during rehabilitation and walking. For cycle testing, MultiTest 2.5-I, ILC 2500 N (load cell), Mecmesin Ltd., Horsham, UK was used. For load failure testing, OmniTest 10, ILC 10 KN (load cell), Mecmesin Ltd., UK was used. The jig used in the experiments had a 40 mm metal head for applying loading to the acetabulum; the hemipelvis was fixed using 10 bolts drilled through the jig to minimize error due to size and rotational variables [36].

**Figure 6:**
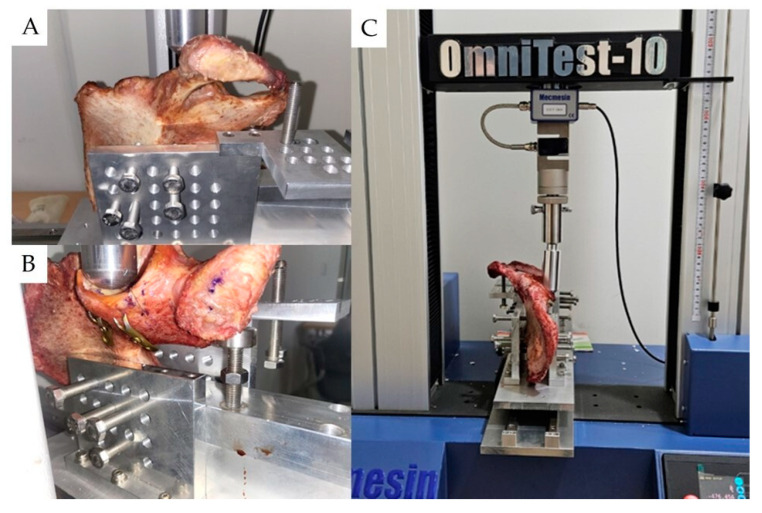
Scheme of experiment. (**A**) The jig was manually adjusted using metal bolts and was fixed to the posterior wall model. (**B**) The jig was manually adjusted using metal bolts and was fixed to the posterior wall model. (**C**) Both quasi-static loading and cyclic loading tests were conducted, with quasi-static loading measuring the force until mechanical failure. The cyclic test was set biomechanically based on a 70 kg subject, with the maximum loading during walking being set at 1400 N. The preload was set at 1000 N for 1000 cycles, followed by 1400 N for another 1000 cycles at a frequency of 1Hz.

For each fixation method, the hemipelvis was fixed to the specific jig, and the jig’s metal head was aligned perpendicularly to the fracture site. Both quasi-static loading and cyclic loading tests were conducted, with quasi-static loading measuring the force until mechanical failure—defined as the point where the compression force and the plate’s buttress force diverged from linearity. (Figure 6) The Vector pro MT program (Mecmesin Ltd., UK) software was used for force measurements [37].

The cyclic test was set biomechanically based on a 70 kg subject, with the maximum loading during walking being set at 1400 N (2.0–3.5 times body weight) [38,39]. The preload was set at 1000 N for 1000 cycles, followed by 1400 N for another 1000 cycles at a frequency of 1 Hz. The EmperorTM program (Mecmesin Ltd., UK) was used for measuring force, stiffness, and displacement during the test. The experiment was concluded when displacement exceeded 2 mm. 

Furthermore, this study also conducted a fatigue study for each type of plate during cyclic testing. Fatigue is defined as the difference in the interfragment gap observed after 100 preloads in a 10,000-cycle test, namely the difference between the gap at 1500N and the gap at free load, which is set as the starting point value. The difference in the interfragment gap at the end of 10,000 cycles is set as the endpoint value. The fatigue of the plate is defined as the difference between the gap at the endpoint and the starting point. 

To minimize bias, subjects with the highest and lowest displacements were excluded from the statistical analysis. In our study, subjects with the highest and lowest displacements were excluded from the statistical analysis to minimize bias. This aligns with standard biomechanics practices, as extreme values can disproportionately influence results. Barnett and Lewis in *‘Outliers in Statistical Data’* recommend excluding such data points if they distort outcomes [40]. Our methodology follows these principles to accurately represent biomechanical effects. 

Data are presented as mean ± standard deviation. Displacement data from different experimental groups were analyzed using one-way ANOVA, with a 95% confidence level indicating significant differences. R statistical programming language (R Foundation for Statistical Computing, Vienna, Austria) version 4.3.1 for Windows, was utilized for statistical analysis.

## 3. Results

**Figure 7:**
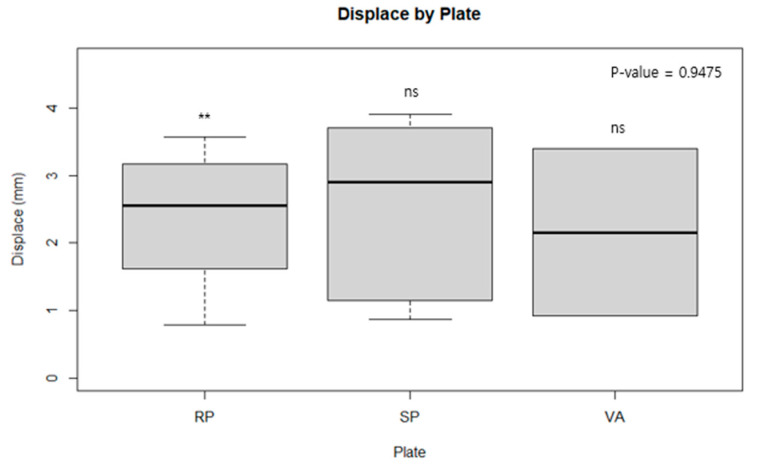
The mean fracture gap of each plate in cyclic test protocol. plates are subjected to a force of 1500 N over 10,000 cycles and the experiment was concluded when displacement exceeded 2 mm. ANOVA analysis revealed no statistically significant differences when comparing the conventional reconstruction plate with the spring plate, the spring plate with the variable angle plate, or when all three were compared together. **, ns: significant at *p*-value ≤ 0.01, or not significant, respectively.

**Figure 8:**
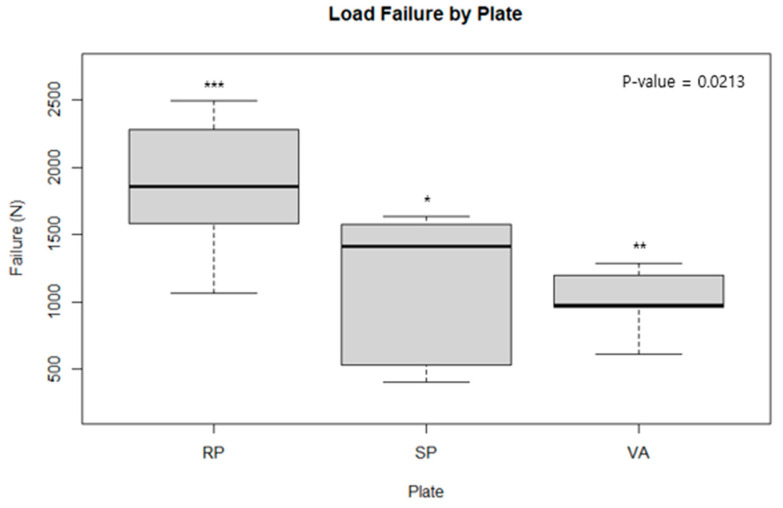
A load failure testing for each type of plate. with quasi-static loading was used, we measured the force until mechanical failure of each plate, i.e., the point where the compression force and the plate’s buttress force diverged from linearity. The conventional reconstruction plate demonstrated significant results against both the spring plate and the variable angle plate. *, **, ***, ns: significant at *p*-value ≤ 0.05, 0.01, 0.001, or not significant, respectively.

**Figure 9:**
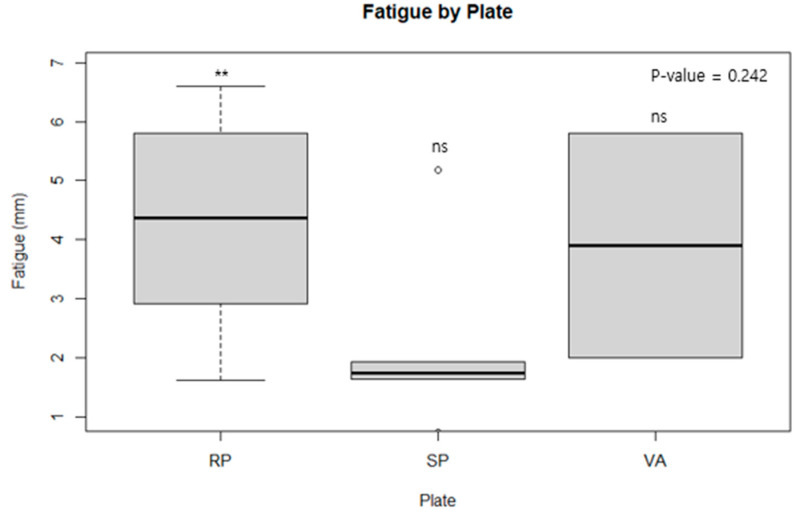
A fatigue study for each type of plate. The fatigue of the plate is defined as the difference between the gap at the endpoint and the starting point. The starting point is the difference between the gap at 1500 N and the gap at free load, while the endpoint is the difference in the interfragment gap at the end of 10,000 cycles and is set as the e value. **, ns: significant at *p*-value ≤ 0.01 or not significant, respectively.

Table 1 displays the mean fracture gap observed in experiments conducted with a 1500 N force according to the cyclic test protocol. ANOVA analysis revealed no statistically significant differences when comparing the conventional reconstruction plate with the spring plate, the spring plate with the variable angle plate, or when all three were compared together. This suggests that, in a cyclic test scenario, where plates are subjected to a force of 1500 N over 10,000 cycles, there is no discernible difference in the ability of the plates to withstand the load (Figure 7). Table 2, however, showed that the conventional reconstruction plate had statistically significant results compared to the other two plates. When each plate was analyzed one-on-one, the conventional reconstruction plate demonstrated significant results against both the spring plate and the variable angle plate, indicating that the stiffness of the conventional reconstruction plate is stronger than the other two. However, no significant difference in stiffness was found between the variable angle plate and the spring plate (Figure 8 and Figure 9).

**Figure 10:**
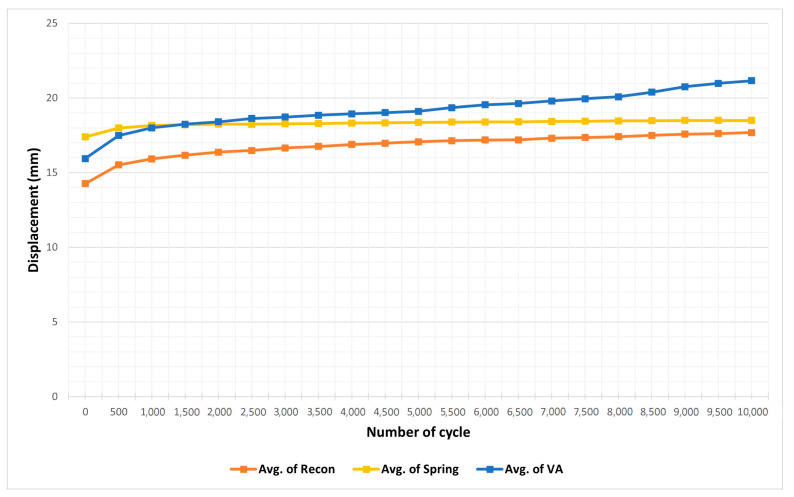
Average displacement of plates during the cyclic test. Each type of plate plate is subjected to a force of 1500 N over 10,000 cycles We compared and analyzed the degree of displacement in 500 units of the cycle. In total, four hemipelves were used for each type of plate, but a failure occurred in one of the models of the spring plate, so the spring plate has only three results.

Statistical analysis of the fatigue values for each plate showed no significant differences among the three types of plates (Figure 10). This indicates that there were no discernible differences in the material properties and design of the plates when subjected to repeated loads of 1500 N over 10,000 cycles. Fatigue results showed no significant differences, and it was decided to proceed accordingly. 

There was a cyclic test failure in one model using the 2.7 mm LCP VA plate out of a total of 12 hemipelvis. An obvious porosity was observed in the evaluation of the surgeon who conducted the experiment. We assumed this to be the cause of the cyclic test failure.

## 4. Discussion

Posterior wall fractures are notably difficult to reduce and the success of surgical outcomes is heavily reliant on the quality of reduction rather than merely on the choice of fixation hardware [41,42,43]. Factors such as marginal impaction, femoral head chondral injury, and labral damage also significantly influence outcomes [44]. This study aimed to evaluate different reduction methods for the most commonly observed type of acetabular fractures. All fracture models were uniformly created and reduced without any instances of screw penetration, with variations solely based on the types of plates and screws used.

The conventional reconstruction plate demonstrated a superior stiffness compared to the spring and variable angle plates, which showed no significant differences between them. Despite using screws of different diameters (2.7 mm for VA and 3.5 mm for spring plates), the strength and footprint size of the plates were comparable, suggesting that further investigation is needed to understand the impact of screw diameter on bone fixation strength.

In a secondary experiment, we subjected the same fracture model and fixation methods to a 10,000-cycle load of 1500 N, mimicking the force exerted by a 75 kg patient standing on one leg [45]. No significant differences were found among the groups, suggesting that under consistent load, the type of fixation does not compromise the stability of the implant during rehabilitation [46]. However, failures in cyclic testing were noted with the variable angle plate in older cadavers with osteoporotic bone, hinting at a potential reduction in fixation strength due to smaller screw diameters [26]. This underscores the necessity for further research into the effects of screw diameter on fixation strength in different bone qualities.

The fatigue analysis from cyclic testing did not reveal any significant differences in gap formation at the fracture site before and after testing, although gaps appeared during the cycles. For instance, the midpoint and endpoint gaps at the 10th and 9990th cycles showed fluctuations, which were statistically analyzed to assess the fatigue resistance of each plate. No significant differences in fatigue were observed, indicating that all plates maintained stability throughout the cycles, an important consideration for postoperative rehabilitation timing.

Furthermore, our study draws comparisons with previous studies such as by F. Pease [28], who evaluated different fixation strategies. Although Pease’s study used Sawbones and showed different outcomes, the findings in our cadaver study suggest the need for additional research on the VA LCP plate, which might outperform traditional lag screws.

Clinical relevance is also supported by studies such as those by Abo Elsoud et al. and Kang et al., which have shown promising in vivo results for the stability of these plates, with most patients achieving union and excellent functional outcomes [47,48]. This is corroborated by our findings from the VA-LCP plate study [26], which also reported successful outcomes without complications.

However, the study faces limitations such as the variability in bone quality and the potential presence of osteoporosis in two hemipelvises, reflective of the aging population in Korea and the advanced age of the cadavers, which may compromise experimental validity. Additionally, the use of a saw to create the fracture model and direct loading via the Jig may not fully replicate clinical scenarios, potentially skewing results towards mechanical rather than clinical outcomes.

Despite these challenges, this study is the first biomechanical evaluation of the 2.7 mm LCP VA plate, providing valuable insights into the biomechanical performance of different fixation methods for posterior wall fractures. This contributes significantly to our understanding of these fractures and aids in improving surgical strategies for these complex injuries.

## 5. Conclusions

In conclusion, our findings demonstrate that while the conventional reconstruction plate exhibited superior stiffness, there were no significant differences in performance under cyclic loading conditions among the three plate types. This suggests that all three surgical options—conventional reconstruction, spring, and variable angle plates—provide sufficient stability for postoperative rehabilitation, assuming that patients avoid excessive activities. This study supports the continued use of the conventional reconstruction plate due to its proven stability, making it a reliable choice for surgical interventions. Additionally, the variable angle and spring plates also proved effective for fragment-specific fixation, ensuring adequate stability for these specific surgical procedures. Overall, each method has its merits, allowing for tailored surgical approaches based on patient needs and specific fracture characteristics.

## Figures and Tables

**Table 1:** The mean fracture gap observed in experiments.

**Group**	**Mean (mm)**	**SD** *	**SEM ^†^**
RP ^‡^	2.37	1.04	0.42
SP ^§^	2.51	1.42	0.63
VA ^∥^	2.16	1.75	1.24
**Group vs. Group**	***p*-value**	**95% CI ^¶^**	
RP:SP	0.87	−1.93–1.67	
RP:VA	0.89	−10.34–10.78	
SP:VA	0.83	−7.47–8.18	

* Standard Deviation; ^†^ Standard error of the mean; ^‡^ Reconstruction plate; ^§^ Spring plate; ^∥^ Variable angle plate; ^¶^ Confidence Interval.

**Table 2:** Load failure testing.

**Group**	**Mean (N)**	**SD** *	**SEM ^†^**
RP ^‡^	1855.47	509.43	207.93
SP ^§^	1111.79	592.6	264.55
VA ^∥^	1007.72	260.85	116.45
**Group vs. Group**	***p*-value**	**95% CI ^¶^**	
RP:SP	0.05	−32.96–1520.31	
RP:VA	0.01	293.98–1401.51	
SP:VA	0.73	−620.57–828.72	

* Standard Deviation; ^†^ Standard error of the mean; ^‡^ Reconstruction plate; ^§^ Spring plate; ^∥^ Variable angle plate; ^¶^ Confidence Interval.

## Data Availability

The data presented in this study are available on request from the corresponding author due to (specify the reason for the restriction).
